# Continuous In-Bed Monitoring of Vital Signs Using a Multi Radar Setup for Freely Moving Patients

**DOI:** 10.3390/s20205827

**Published:** 2020-10-15

**Authors:** Sven Schellenberger, Kilin Shi, Fabian Michler, Fabian Lurz, Robert Weigel, Alexander Koelpin

**Affiliations:** 1 Institute of High-Frequency Technology, Hamburg University of Technology, 21073 Hamburg, Germany; fabian.lurz@tuhh.de (F.L.); alexander.koelpin@tuhh.de (A.K.); 2 Institute for Electronics Engineering, Friedrich-Alexander University Erlangen-Nürnberg, 91058 Erlangen, Germany; kilin.shi@fau.de (K.S.); fabian.michler@fau.de (F.M.); robert.weigel@fau.de (R.W.)

**Keywords:** continuous wave radar, remote sensing, vital signs, vital parameter measurement, bed exit detection

## Abstract

In hospitals, continuous monitoring of vital parameters can provide valuable information about the course of a patient’s illness and allows early warning of emergencies. To enable such monitoring without restricting the patient’s freedom of movement and comfort, a radar system is attached under the mattress which consists of four individual radar modules to cover the entire width of the bed. Using radar, heartbeat and respiration can be measured without contact and through clothing. By processing the raw radar data, the presence of a patient can be determined and movements are categorized into the classes “bed exit”, “bed entry”, and “on bed movement”. Using this information, the vital parameters can be assessed in sections where the patient lies calmly in bed. In the first step, the presence and movement classification is demonstrated using recorded training and test data. Next, the radar was modified to perform vital sign measurements synchronized to a gold standard device. The evaluation of the individual radar modules shows that, regardless of the lying position of the test person, at least one of the radar modules delivers accurate results for continuous monitoring.

RFradio frequency CWcontinuous-wave FMCWfrequency-modulated continuous-wave UWBultra-wideband LNAlow noise amplifier LFlow frequency VCOvoltage-controlled oscillator SDstandard deviation Iinphase Qquadrature SNRsignal-to-noise ratio EIRPequivalent isotropically radiated power ISMindustrial, scientific and medical TFMTask Force Monitor ADCanalog-to-digital converter SPIserial peripheral interface UDPuser datagram protocol IBIsinterbeat intervals HSMMhidden semi-Markov model VPvital parameters ECGelectrocardiogram PUTperson under test BrPMbreaths per minute ICGimpedance cardiography ZCzero crossing RMSEroot mean square error

## 1. Introduction

Complications and sudden deterioration are often unexpected in hospitals. In retrospective analyses of deaths in hospitals, less than 30% were expected and compensated by intensive care, and more than 70% occurred unexpectedly [1]. Continuous monitoring can contribute to preventing sudden deaths and achieve a more successful outcome through early response. Several alterations of vital parameters, which indicate potentially serious diseases, occur only irregularly and thus elude momentary assessments [2,3]. Detection of changes in vital parameters can help in risk stratification and subsequently lead to tailored levels of medical monitoring according to individual needs [4] and early intervention in case of deterioration [5,6,7]. Automated registration and analysis of vital parameters based on predefined thresholds enables continuous health monitoring with affordable means and early diagnosis of sporadic diseases [8].

To date, conventional vital sign monitoring is based on a touch-dependent sensor technology, e.g., heartbeat by electrocardiogram (ECG), pulse rate and oxygenation of the blood by pulse oximetry. Using this type of sensor technology, mobility and autonomy are significantly limited for patients in need of care. In addition, skin irritation can occur when using adhesive electrodes and the constant unaccustomed feeling caused by sensors and cables can lead to discomfort and manipulation of the sensors, false alarms and an increased workload for clinical staff. A large collective of patients would profit from an adaptable and non-invasive form of monitoring [9], especially if this kind of monitoring could be touchless and reduce the workload for staff.

An emerging possibility to measure vital parameters from the distance is using radar technology. For the most part, either the relative or absolute distance to a person’s body is measured and this information is further evaluated. On the one hand, breathing causes the volume of the thorax of a person to increase and decrease and on the other hand, heartbeat causes a pulse wave that spreads along the vessels and becomes visible on the skin surface [10,11]. In addition, it has been found that, similar to the stethoscope, heart sounds can be measured by small vibrations on the chest in the micrometer range [12]. The use of radar to monitor heartbeat and respiration has been investigated by several research groups with promising results. In the process, different approaches and radar prototypes with different frequencies and modulations have been developed. For example, continuous-wave (CW) Doppler radars at 2.4GHz [13,14], 5.8 GHz [15] and 24 GHz [16,17] were built and used to measure respiration, heartbeat, or motion activity. Furthermore, frequency-modulated CW systems at 77 GHz [18,19] and 122 GHz [20] are used to measure vital parameters of several people simultaneously by separating the targets. In addition, ultra-wideband radars with different frequencies and bandwidths are used to measure vital parameters [21,22,23].

Random body movement cancellation methods are also investigated to remove unwanted motion artifacts and retrieve vital parameters even during larger movements [24,25,26]. However, it has been found that due to a large movement the signal is already distorted by hardware intermodulation before digitization in such a way that certain components for vital parameter detection are masked, so that the compensation is not easily possible later on [27].

In this work, we present a signal processing method which can automatically detect sections in the raw data without artifact movements of the test person in order to achieve a reliable vital parameter detection in the remaining sections. This signal processing routine for the monitoring of freely moving patients is introduced and evaluated. Furthermore, using this method, the raw data of the four 24 GHz CW radar modules are divided into the four states: “Absence”, “resting”, “bed exit”,” bed entry”, and “on bed movement”. For the first time, the performance of the individual modules at different lying positions using a synchronised gold standard device is examined.

## 2. Measurement Setup and Protocol

The radar system used for vital sign monitoring was designed to be installed in hospital beds. With its four radar modules it covers the entire width of a bed, so that patients can position themselves freely within the bed. In this section, the structure of the radar system will be discussed in more detail. In addition, an adjustment to the system has been made in order to carry out recordings with the radar simultaneously to a gold standard reference device. Finally, the measurement protocol for recording the labeled data, on which the presented algorithms are based, is discussed.

### 2.1. Multi Radar System

**Figure 1:**
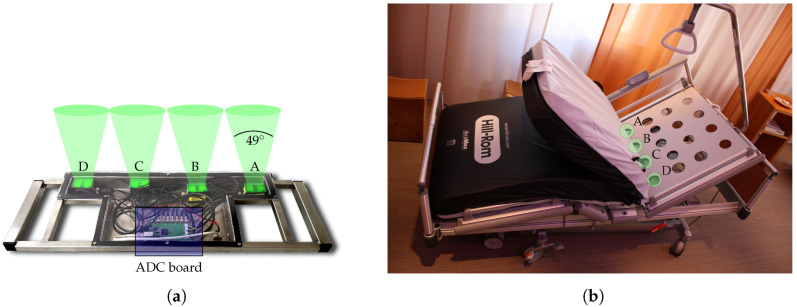
Photographs of (**a**) fabricated radar system prototype and (**b**) hospital bed with system installed.

As mentioned above, the multi radar system is intended for coverage of beds. The system is placed under the bed frame and measures the person under test (PUT) through a foam mattress. A photograph of the prototype is shown in Figure 1a. The entire electronics are mounted in a stainless steel frame which is sealed by a *Makrolon* polycarbonate panel. As illustrated in the picture, the system consists of four separate modified radar modules of the type *iSYS-4001* from *InnoSenT*. The modules are CW radars with different operating frequencies within the 24 GHz ISM band in order to prevent crosstalk. They also have a spacing of 18 cm to each other. In Figure 1a the antenna beams of the bistatic setup are indicated. The half-power beamwidth is 34° and 49°, respectively. The permittivity change between air and the 18 cm thick foam mattress is negligible. Therefore, the mattress is effectively transparent for the radar modules. Moreover, two SMA connectors were integrated into the housing of the individual modules to directly capture the down-converted inphase (I) and quadrature (Q) signals. For simultaneous sampling of the eight raw signals, the analog-to-digital converter (ADC) *ADS1298* from *Texas Instruments* with eight channels is used. It provides a resolution of 24 Bit and a sampling rate of 2000 Sa/s. The digitized data are streamed in user datagram protocol (UDP) packets via Ethernet to a connected PC using an *Infineon XMC4500* microcontroller. A hospital bed with integrated radar is shown in Figure 1b. The positions of the individual modules are indicated by green circles and are labeled using the same letters as in Figure 1a. As can be seen in the picture, the modules are spaced to make use of the holes in the bed frame of the hospital beds at the palliative care station of the university hospital in Erlangen. All four holes are equipped with radar modules to provide redundancy to measure the person regardless of which side of the bed they are lying on. Additional information on the system can be found in [28].

### 2.2. Reference Device

**Figure 2:**
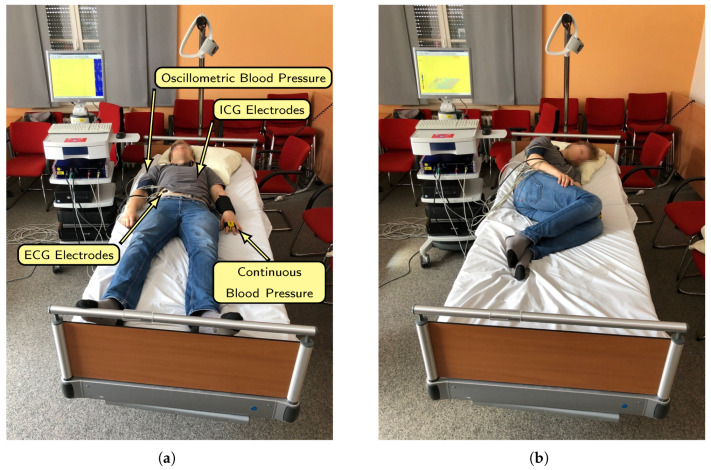
(**a**) A photograph of a test subject in supine position wired to the reference device. The different sensor locations are indicated. (**b**) Test subject rotated on the left side.

To evaluate the performance of the individual modules of the system, a reference device for synchronous measurement of respiration and heartbeat is used. The so-called Task Force Monitor (TFM) from *CNsystems Medizintechnik* allows synchronous recording of ECG, impedance and continuous blood pressure. Additionally, the TFM offers the function to sample external signals simultaneously. All attached sensor locations are roughly indicated in Figure 2a. First of all the six channel ECG has four spot electrodes that are attached to the upper body. For the impedance measurement one band electrode is placed at the neck, two band electrodes under the chest and one spot electrode on the left leg. In addition, a blood pressure cuff is used to determine the oscillometric blood pressure and, in combination with the finger cuff, the continuous blood pressure. The blood pressure sensors are only attached for the purpose of a full setup, the blood pressure is not needed for evaluation.

To synchronize radar and TFM data, a synchronization sequence based on the Gold codes is generated within the microcontroller of the radar which is then sampled by the radar and the TFM. After the measurement, the shift between the two sequences can be determined and corrected using autocorrelation. In this way a very precise synchronization can be achieved. More information on the synchronization procedure can be found in [29].

### 2.3. Measurement Setup and Protocol

**Figure 3:**
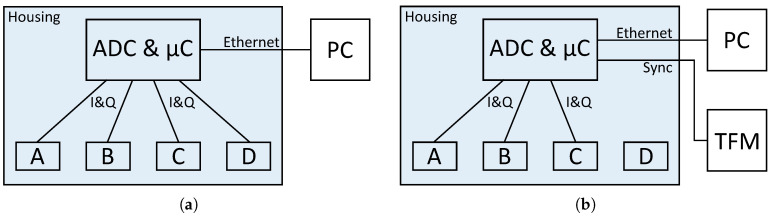
Block diagrams of the measurement setup (**a**) for movement classification and (**b**) for vital sign evaluation.

In the following section the different measurement setups and protocols are described. There are two setups, because the system has to be modified to synchronize it with the TFM. Therefore, the measurements for the vital sign evaluation per module were performed separately. All other measurements were performed with the actual configuration to come as close as possible to the scenario in the hospital. Block diagrams of both setups are shown in Figure 3, they will be discussed below.

#### 2.3.1. Presence and Movement Classification

The first important step before evaluating the vital parameters is to classify presence and movement. For this reason, various radar measurements have been made in which the subjects enter, exit or move on the bed. The radar is used in the same way as it is used in the hospital. A block diagram of the configuration is shown in Figure 3a. Instead of connecting the radar to the hospital server it is connected to a local PC for the duration of the measurements. These measurements are done without the reference device, since various movements such as leaving the bed on different sides or lying on the chest are not possible with wired sensors attached to the thorax and arms.

The protocol requires the test persons to turn from a supine position to the right side, then to prone and finally to the left side in the first measurement. Between the rotations, the test persons lie on the bed for two minutes in a relaxed position. The rotations are performed twice in total. Next, measurements were made in which the subjects enter the bed a total of three times and then exit the bed again. Between actions there was a pause of at least 10 s. During one measurement the bed was entered from the right side and during another one from the left side. Finally, a measurement was recorded which serves as test dataset in order to evaluate the performance of the algorithm. Hereby the test persons enter the bed after 15 s and rotate through the four lying positions with a 30 s pause in between. Then, the subjects leave the bed again while recording continues for another 15 s.

#### 2.3.2. Vital Sign Evaluation

A comparison of the multi radar system with the reference is intended to show the performance of the individual modules in order to learn how to combine the results of the individual modules. Furthermore, the effectiveness of the multiple radar modules can be tested at different lying positions. For these measurements an output of the microcontroller is connected to the external input of the TFM. A block diagram of the setup is shown in Figure 3b. Given that the synchronisation sequence must also be sampled by the system’s eight channel ADC, which is already fed by the eight signals from the radar modules, one of the modules cannot be connected for these measurements. Since modules B and C are expected to give better results and module D is located at the edge of the bed, the synchronisation sequence was connected to one of the channels of module D. Therefore, only three radar modules are used for the vital sign measurements.

The measurement protocol for this scenario consists of three lying positions. First, the test person lies in a supine position, then on the left side and finally on the right side. Because the TFM’s electrodes are attached on the stomach, it is not possible to take measurements lying on the stomach. In all positions data are recorded for five minutes. In the beginning of each measurement the subject does a small calibration movement for an ellipse fit that is done later on in the data evaluation. Therefore, the first 20 s are ignored for the further evaluation of vital signs.

## 3. Radar Signal Processing

**Figure 4:**
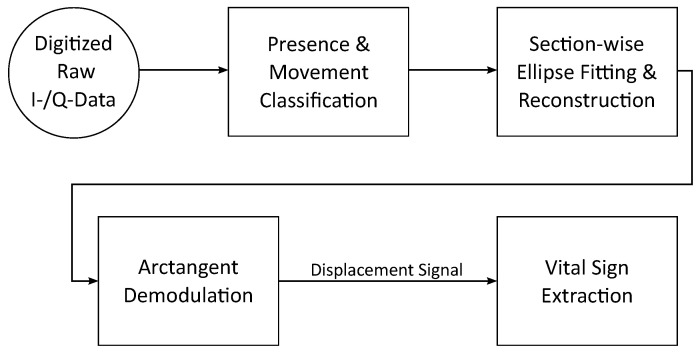
A flowchart showing the overall signal processing steps of the proposed algorithm.

The overall processing steps for continuous monitoring of vital signs including detection of bed exits and entries is shown in Figure 4. At the beginning, the raw data of the four radar modules are digitized. Then, based on the raw data, sections in which a person is present are detected and movements are classified. In the next step, an ellipse fit for distance reconstruction is performed for each section with a person present to compensate for non-idealities. Then, relative distances of the individual systems are calculated from the phase using arctangent demodulation. Last, the resulting displacement signals are further evaluated to retrieve heartbeat and respiration information. In the following all steps will be explained in more detail.

### 3.1. Presence and Movement Classification

Before the digitized raw data of the radars are reconstructed, the first step is to detect sections where a person is actually present. Within these segments stronger movement is to be differentiated from resting phases where vital signs can be determined more reliably. This pre-processing is intended to simplify the ellipse fit later and gives information about when a person was in bed and how much this person was moving.

As shown before in [30] the presence detection using radar data can be achieved by using a support-vector machine. Since there is insufficient data available to train a classifier in this case, only one feature, the standard deviation (SD), is used with an empirically determined threshold value. For the presence determination all raw channels are split in windows of 2 s of data. Then, the SD is calculated according to:

(1) $$\mathrm{SD}=\sqrt{\frac{1}{N-1}\sum_{n=1}^{N}{\left|X_{n}-\bar{X}\right|}^{2}},$$

with *N* as the length of the sample vector *X*, for every window. A section is then classified with a person present when the SD of more than four out of eight channels is larger than 0.7 mV.

During segments of heavy movement, vital parameters are difficult and unreliable to determine. Therefore, the next step is to detect increased movement in order to process only sections with calm breathing. These movements can then be divided into different classes to obtain additional monitoring information. Two features are used to differentiate between smaller and greater motion, which are assessed with an empirically determined threshold value, as with the presence detection before.

For the calculation of the features the raw signals are pre-processed. Each signal is normalized to the first measured value and then the absolute values of the first derivative are determined. In this way it is possible to get information about how much the signal has changed between adjacent samples. Similar to the presence detection, the signals are divided into 2 s windows and then the two feature values are calculated for each window. First of all, the variance of the windows is calculated using:

(2) $$\mathrm{Var}=\frac{1}{N-1}\sum_{n=1}^{N}{\left|X_{n}-\bar{X}\right|}^{2}.$$

In addition, the second feature is determined in which the absolute value of the difference of the mean value of adjacent windows is calculated. This feature indicates the average change between two seconds of data. If the variance of a window is larger than 4 × 10^−8^ or the average change between two windows is 4 × 10^−4^ then the window is classified containing movement. However, a 2 s section of the total data is only classified as movement if the signals of all radar modules indicate strong movement.

**Figure 5:**
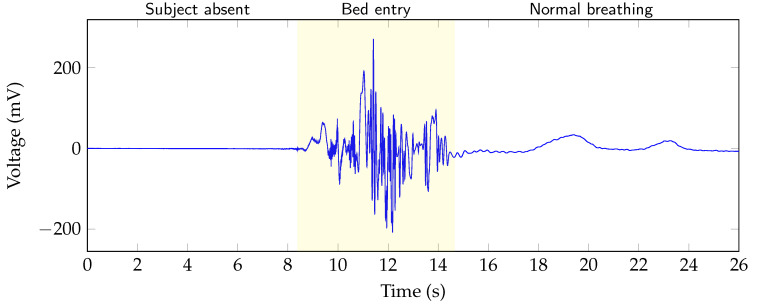
An exemplary section of the raw I-channel data of one of the radar modules from a test measurement. The bed entry section is highlighted.

After presence and motion is detected in the raw data, the information can be combined to specify the motion more precisely. By combining the order of the two states, movements can be further categorized into three classes. These classes are: “bed entry”, “bed exit” and “on bed movement”. In Figure 5 the raw data of a channel are shown. The different areas “subject absent”, “bed entry” and “normal breathing” are highlighted in the figure. In case of the data in the figure, it can be concluded that a large movement after a section of absence must be a bed entry. Similarly, a movement following absence can be classified as a bed exit and if a person is present before and after the movement, this person has moved on the bed. For further processing, the different states are coded by using a state vector. Sections where no person is in bed are marked with the state 0. When a person is lying calmly in bed, the state changes to 1. If the person is moving in bed, the state changes to 4. However, a bed entry and exit is marked with 2 and 3, respectively. Table 1 gives an overview of the different states. Using this information, the ellipse fit can then be applied in a selective way.

### 3.2. Section-Wise Displacement Signal Reconstruction

Ideally the raw data of a CW radar represent a circle centered around the origin in the I/Q domain, if the target moves with a constant velocity. However, non-idealities of the components induce gain errors that distort the circle into an ellipse. Furthermore, offset errors lead to a shift of the circle center from its ideal position. During a strong movement the position and also the angle of the target can change, which also causes further distortion of the raw data. Due to the change of the target, it is not possible to reconstruct continuous vital parameters and therefore these sections are unusable for further analysis. With the obtained information about usable sections in the raw data using the presence and movement classification, a section-wise ellipse fit and distance reconstruction can be performed for the data of each radar module separately. The next steps are only performed on sections consisting of state 1, which corresponds calm motions.

First an ellipse fit is conducted on the raw data with an algorithm based on [31], then the data are normalized to the unit circle using the extracted parameters. Next, the relative displacement d(t) can be determined, according to theory [32], from the phase differences φ(t) between transmitted and received signal

(3) $$d\left(t\right)=\frac{\varphi (t)}{4\pi }\times \lambda_{0},$$

using the module related wavelength $\lambda_{0}$. The phase change over time can be calculated from the reconstructed I(t) and Q(t) signals of the radars

(4) $$\varphi \left(t\right)=\arctan\left(\frac{Q(t)}{I(t)}\right).$$

In the last step the vital signs are extracted from the reconstructed displacement information.

### 3.3. Vital Sign Evaluation

The demodulated displacement signal reflects the distance change between radar and PUT. Considering a PUT at rest, the distance signal consists of a combined motion due to respiration and cardiac activity. For the evaluation of the respiratory rate and heart rate, the individual components must be separated from each other. The different steps are explained in the following sections.

#### 3.3.1. Respiration Signal Analysis

Taking the respiratory rate of a healthy person of 10...25 breaths per minute (BrPM) into account, the cutoff frequencies of the fourth order Butterworth bandpass filter are selected at 0.07 Hz and 0.7 Hz to a corresponding range of 4...40 BrPM [33,34]. After filtering the displacement signal, all zero crossings from positive to negative values of the respiration signals are determined. The same processing steps are also done for the reference respiration signal, which is derived from the impedance signal of the TFM. To compare the results, the durations of the consecutive breaths are interpolated and sampled in one second intervals. Before interpolation the values are smoothed with a median filter of size 5 and a smoothing filter of size 6, both Matlab internal functions, to compensate for outliers. Then, the root mean square error (RMSE) is calculated using the equidistant sampled predicted and reference respiration intervals *I* and $I_{Ref}$:

(5) $$\mathrm{RMSE}=\sqrt{\frac{\sum_{n=1}^{N}{\left(I_{n,Ref}-I_{n}\right)}^{2}}{N}}.$$

#### 3.3.2. Heartbeat Signal Analysis

The vibrations of the chest caused by the heartbeat can be separated into the pulse waves [10] and the heart sounds [12] by choosing the right cutoff frequencies. Furthermore, Will et al. stated that the evaluation of heart sounds allows for a more precise detection of heartbeats. Therefore, the heart rate is determined according to [12] using a heart sound segmentation algorithm based on a hidden semi-Markov model (HSMM). The HSMM predicts the different states of the heart sound as: first heart sound, systole, second heart sound, and diastole. From these states the interbeat intervals (IBIs) can be extracted, which constitute the distances between successive heartbeats. Moreover, the IBIs are also determined from the ECG by calculating the differences between successive R-peaks. The following steps for evaluation are identical to the respiration. The IBIs are interpolated and sampled equidistant in steps of seconds and then the RMSE is determined for performance comparison by using Equation (5).

## 4. Results and Discussion

Following the description of the measurement setup and signal processing routine, the next section describes the measurement results, starting with the presence and movement classification. Then, an evaluation of the vital sign detection performance on a small number of subjects is presented. An overview of the subjects and the performed measurements is given in Table 2.

### 4.1. Presence and Movement Classification

**Figure 6:**
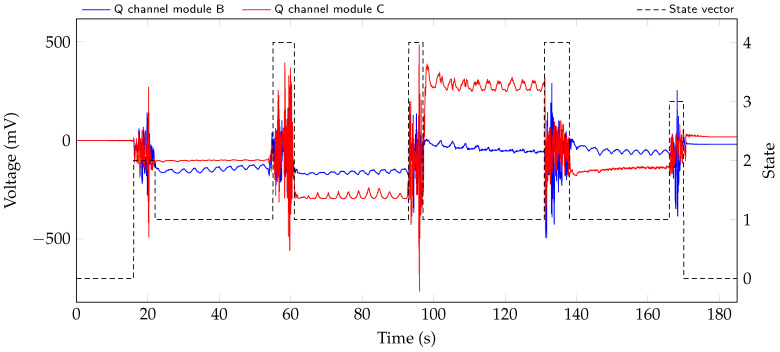
Two of the eight raw signals during the test scenario with resulting state vector in black.

For presence and movement classification, data was recorded according to the measurement protocol with two test persons. In a first step the thresholds for the algorithm described in Section 3.1 are determined from the different scenarios. Then, the classification is applied to the test dataset that was recorded. The resulting state vector for the measurement of subject 1 is shown with raw signals of radar modules B and C in Figure 6.

The larger movements at 18 s, 58 s, 95 s, 135 s, and 167 s are all correctly detected and classified. In addition, the sections at the beginning at around 0... 16 s and end from about 170... 185 s of the measurement are correctly marked as no person present in the bed.

In the next step, the reference labels as well as predicted states for both test datasets are windowed in sections of 5 s. Each window is given the value of the most commonly occurring state. Corresponding windows are then compared with each other and visualized using a confusion matrix. As can be seen in the resulting confusion matrix, which is shown in Table 3, almost all predicted states correspond to the reference. The two “bed entries” and “ bed exits” and six “on bed movements” are correctly detected. There is a misclassification, as there is an “on bed movement” window predicted as a calm section. Which can be explained through a longer predicted movement state and the windowing process. An overall accuracy of 98.6 is achieved.

These results show that it is possible to identify presence and movement within the raw signals of the radar using simple and low computational features. More annotated data to test the algorithm is desirable. Furthermore, more training data could be used to test an approach based on machine learning to avoid an empirical threshold.

### 4.2. Vital Sign Extraction

Taking a look at the raw signals in Figure 6, it is possible to see the respiration in the signals. Comparing the amplitude change due to respiration in the four sections with state 1, it can be seen that, depending on the lying position of the test person, the respiration is measured with varying accuracy by the two systems.

**Figure 7:**
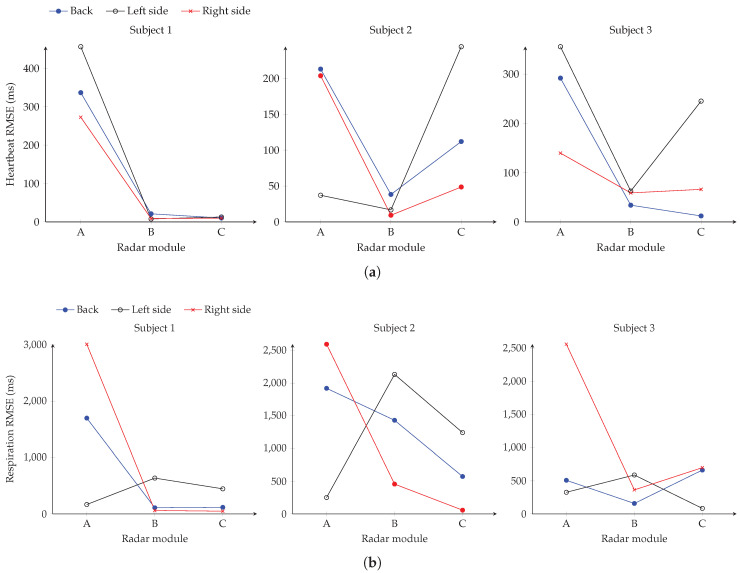
Heartbeat (**a**) and respiration (**b**) RMSE results of the three radar modules in different lying positions for the three subjects.

The data for evaluating the performance of the individual modules was recorded according to the measurement protocol with all subjects listed in Table 2. In the next step, the performance of the single modules is determined in various lying positions compared to the reference device. In Figure 7 the RMSEs of heartbeat and respiration evaluation for all subjects in the three lying positions are shown for the three radar modules. Considering the error in Figure 7a of the heartbeat analysis with the patient lying on his back, it can be seen that especially modules B and C perform well in all subjects. This is due to the fact that both modules are at the appropriate height to the heart in this position. Therefore, errors of about 10 ms, 38 ms and 12 ms are obtained for the three subjects, respectively. In addition, it is noticeable that after the rotation of subject 2 to the left side, particularly the result of module A changes. The RMSE of module A reduces to 37 ms, but module C is considerably worse with a value of 244 ms. Module B still seems to have the better spot on the upper body and delivers the best performance with 16 ms. This could be explained with the subject being mainly above modules A and B after rotation. In general, subject 2 and 3 show a position-dependent change in the output of the modules. Only for subject 1 the results remain constant. This can be explained by the positioning of subject 1, who continues to occupy a similar position in the bed after rotation. However, regardless of the subjects lying position in bed, there is at least one radar module that provides a suitably small RMSE for continuous heartbeat monitoring.

Observing Figure 7b, it can be seen that the performance of the individual modules is different for the respiration evaluation than for the heartbeat. Considering subject 1, modules B and C have a low RMSE of 108 ms and 116 ms while the subject is lying on his back. Also on the right side both modules have a deviation of 47 ms and 59 ms. In comparison, the value of module A is lowest with 166 ms when the person is lying on the left side. In contrast, the results of subject 2 are slightly worse. When measuring on the back, the best module has an RMSE of 572 ms which can be explained partly due to the low breathing rate between 6...12 BrPM of the subject. For the measurement position on the left side, module A has the smallest error of 250 ms and module C lying on the right side a value of 56 ms. In contrast, module C delivers the lowest error of 82 ms for subject 3 in the lying position on the left side. However, it can be seen that module B delivers the best results in the other scenarios for this test person. Also with the respiration evaluation it is noticeable that there is at least one radar module, which supplies data with a small error in comparison to the reference. Overall, the radar system is well suited for continuous monitoring of vital signs in a bed, considering the similar anatomy of the subjects. The low variability of the anatomy does not allow conclusions to be drawn about the performance in persons with a different height, more deviating BMI or from other age groups. The design with several modules creates a redundancy so that at least one module provides a medically useful result for heartbeat and respiratory analysis. During usage in a real-life application without reference devices, an automated selection of the best module as described in [35] could be implemented. Which of the modules delivers the best result seems to depend largely on the lying position in the bed. However, the best modules for respiration and heartbeat detection may differ. In a future step, the results of the individual modules must be combined so that only one value with a minimum error is determined. In addition, an evaluation with test persons of different heights would be of interest, as the measurement position along the upper body could change.

## 5. Conclusions

In this article, a system based on multiple radar modules for continuous in bed monitoring of vital sings was presented and compared to a gold standard reference device. The radar system was tailored for hospital beds and modified for a synchronization with the reference device. In order to simulate a realistic scenario in the hospital, measurements were made with two test persons to obtain labeled movements. Subsequently, measurements were taken with three subjects and a reference device in different lying positions. The measurements for movement and vital parameters were performed separately, as sufficient movement wired to the reference device was not possible. Then a signal processing routine was presented to divide sections of the raw data into the classes “absence”, “resting”, “bed entry”, “bed exit”, and “on bed movement”. This pre-processing of the raw signals allowed the identification of sections where the person lied calmly in bed. Finally, heartbeat and respiration can be determined more reliably in these resting areas. Using the separately conducted reference measurements, the performance of the individual radar modules in different lying positions was evaluated. The results show that in each position at least one module allows for a valid vital parameter detection. Selecting the best module by hand, respiration detection achieved in the best measurement an RMSE in comparison to the reference of 47 ms, with a mean error over all measurements of 200 ms. In comparison, the heartbeat detection achieved an error of 8 ms during the best measurement and 24 ms on average over all measurements. In the future, a possible combination or selection of the results of the individual modules should be investigated. Moreover, a study with a larger number of test persons is reasonable for testing the algorithm.

## Figures and Tables

**Table 1:** Possible states that can be assigned by presence and movement classification.

State	Description
0	No person present
1	Calm movement
2	Bed entry
3	Bed exit
4	On bed movement

**Table 2:** Overview of all subjects.

#	Age	Sex ${}^{1}$	Height (cm)	Weight (kg)	BMI ${}^{2}$	Movement Measurements	Vital Sign Measurements
1	29	M	183	75	22.4	x	x
2	28	M	187	85	24.3	x	x
3	28	F	175	79	25.8		x

${}^{1}$ M: male, F: female; ${}^{2}$ Body mass index ( kg/m2).

**Table 3:** Confusion matrix for the resulting state vectors of both test datasets.

		Predicted State				
		0	1	2	3	4
**True State**	**0**	11	0	0	0	0
	**1**	0	50	0	0	0
	**2**	0	0	2	0	0
	**3**	0	0	0	2	0
	**4**	0	1	0	0	6
